# Self-Prescribed Dietary Restrictions are Common in Inflammatory Bowel Disease Patients and Are Associated with Low Bone Mineralization

**DOI:** 10.3390/medicina55080507

**Published:** 2019-08-20

**Authors:** Tiziana Larussa, Evelina Suraci, Raffaella Marasco, Maria Imeneo, Ludovico Abenavoli, Francesco Luzza

**Affiliations:** Department of Health Sciences, University of Catanzaro “Magna Graecia”, 88100 Catanzaro, Italy

**Keywords:** inflammatory bowel disease, diet, patient-centered care, bone mineralization, nutritional status, health behaviors, public health

## Abstract

*Background and objectives:* Despite the serious concerns of patients about the role of food in triggering or ameliorating their intestinal disease, there are few studies dealing with patients’ beliefs and practices regarding diet in inflammatory bowel disease (IBD). The aim of this study was to investigate how the disease affected the dietary habits of patients with IBD, and to assess if patients’ food restrictions were responsible for low bone mineralization. *Materials and Methods:* For this study, 90 consecutive patients referred for IBD were interviewed regarding their dietary habits. Demographic features and clinical characteristics potentially associated with the dietary habits were collected. A validated and self-administered survey questionnaire dealing with dietary habits and patients’ beliefs and perceptions regarding food was analyzed. Multivariate logistic regression analysis was performed in order to identify risk factors for dietary restrictions among participants and to evaluate the relationship between dietary restrictions and low bone mineral density (BMD). *Results:* Among the 63 (70%) patients who claimed a self-prescribed dietary restriction, 84% avoided dairy products. Significant risk factors (adjusted odds ratio (OR), 95% confidence interval (CI)) for the dietary restrictions were a younger age (*p* = 0.02), a higher level of education (*p* = 0.007), and a higher visceral sensitivity index (*p* = 0.009). Most (80%) of the patients displayed an inadequate calcium intake, and an abnormal result at dual-energy X-ray absorptiometry (DXA) scan accounting for low BMD was reported in 46 (51%) of them. Dietary restrictions (*p* = 0.03), and in particular avoiding dairy products (*p* = 0.001), were significant risk factors for a low BMD, along with female gender (*p* = 0.001), smoking (*p* = 0.04), and steroid abuse (*p* = 0.03). Almost all (86%) patients changed their diet after IBD diagnosis, as 8% believed that foods could have been a trigger for IBD and 37% that a proper diet was more important than drugs in controlling disease. Although 61% of the patients claimed to have received nutritional advice, 78% of the participants showed interest in receiving more. *Conclusions:* Dietary habits of IBD patients should be investigated by healthcare professionals as part of the routine visit. Clinicians are invited to provide nutritional recommendations to these patients in order to avoid unnecessary self-prescribed dietary restrictions.

## 1. Introduction

Crohn’s disease (CD) and ulcerative colitis (UC) are the main clinical phenotypes of inflammatory bowel disease (IBD), both characterized by a condition of chronic relapsing intestinal inflammation [1]. Although an association with immune system dysregulation, intestinal microflora, and environmental factors has been strongly suggested, the etiology and pathogenesis are still not fully elucidated [2,3,4]. Nonetheless, current therapeutic goals are aimed at inducing and maintaining remission, using anti-inflammatory drugs as well as immunosuppressant and biological agents [5]. The so-called biologic era, based on drugs such as infliximab and other anti-tumor necrosis factor monoclonal antibodies, represents a revolution in the treatment scenario of IBD and novel biologic agents are still emerging [6]. Nevertheless, safety concerns limit their use, and also 20–30% of patients fail to respond, thus leading clinicians towards the optimization of this therapy [7,8].

Patients with IBD see in diet modification a potential treatment to manage their illness, and most of them believe that diet has a role in the disease pathogenesis, or dietary factors could cause a relapse in the illness. Therefore, up to 68% of IBD patients limit their diet in trying to control symptoms and avoid exacerbation of the disease [9]. IBD patients have frequently reported that the disease affects both the appetite and the pleasure related to eating, to the extent that dietary beliefs and behavior have a strong impact on their social life [10]. The trend among patients is to seek dietary advice from the Internet or support groups, rather than referring to their healthcare team. A common consequence of this is the adoption of self-prescribed exclusion diets, mostly lacking any strong scientific evidence in the IBD setting, such as a specific carbohydrate-based diet (SCD), paleolithic diet, anti-inflammatory diet (inflammatory bowel disease anti-inflammatory diet, IBD-AID), gluten-free diet (GFD), and low FODMAP (fermentable oligo-, di-, monosaccharides and polyols) diet [11]. Herfarth et al. reported that 65.5% of IBD patients on a GFD showed improvement of at least one clinical symptom, but there is no evidence regarding the usefulness of GFD in maintaining IBD remission [12]. Similarly, potential benefits from SCD, IBD-AID, and low FODMAP diet in IBD patients are still under investigation, but limited data are available with regard to their efficacy [13,14].

Studies conducted on dietary behavior in the IBD population have reported that self-prescribed dietary regimen is common among IBD patients, as an attempt to ameliorate symptoms and prolong remission intervals [15]. Although some foods are recognized as a trigger for the induction of bowel symptoms in IBD patients, an indiscriminate exclusion of certain foods can lead to serious nutritional deficiencies, with consequent malnutrition increasing the risk of hospitalization [16]. In particular, milk and dairy products, which represent the main source of calcium and vitamin D, are the most frequently avoided foods by patients, especially during IBD flares [17,18]. According to both the United States Department of Agriculture (USDA) [19] at the international level, and the Reference Levels of Nutrients and energy intake (LARN, Nutrients and Energy Reference Levels) [20] at the Italian national level, the current recommended dietary allowance (RDA) for calcium varies between 700 and 1200 mg/day throughout a person’s life. Daily intake of calcium below the recommendations is seen more often and vitamin D levels have been found insufficient both in CD and UC patients [21,22]. The biggest concern that follows is the adverse effect on bone health, as nutritional consequences of dairy product restriction might impact negatively on bone mineral density (BMD) [23]. Moreover, IBD is already a condition associated with bone mineral loss, development of osteoporosis, and increased risk of fracture, with the underlying inflammatory process, as well as the low body mass, corticosteroid use, and malabsorption, all commonly recognized factors in contributing to bone disease [24]. Therefore, unnecessary dietary restrictions such as dairy products containing calcium and vitamin D should be avoided for these patients. The aim of this study was to investigate the prevalence, risk factors, and motivation of self-prescribed food restriction in a series of IBD patients, and evaluate the association with low BMD.

## 2. Materials and Methods

### 2.1. Study Design and Patient Recruitment

Over a three-month period, a single-center cross-sectional study was conducted at the Digestive Pathophysiology Unit of the University Hospital in Catanzaro, in Southern Italy, to investigate the self-prescribed dietary restrictions in patients with IBD who were consecutively admitted to the outpatient clinic. The aim of the study was to collect data on the prevalence of dietary restrictions and evaluate the association with bone demineralization in a series of IBD patients.

### 2.2. Eligibility Criteria

To be eligible for the study, patients were required to be older than 18 years of age, to have a documented diagnosis of CD or UC based on clinical, radiological, and endoscopic criteria for at least 12 months, and provide a BMD assessment and vitamin D serum levels within the preceding 12 months. Patients were considered not eligible when presenting a short bowel syndrome, responsible for nutrient malabsorption, or having alterations in calcium metabolism of primitive origin, such as primary hyperparathyroidism, which independently could determine alterations in BMD. All patients had to be able to provide written informed consent.

### 2.3. Ethics and Consent

The study protocol was approved by the University of Catanzaro Ethics Committee (n.86/2019). All participants provided informed written consent prior to participating in the study.

### 2.4. Data Collection

Patient characteristics, medical history, lifestyle habits, and medications were extracted from the patients’ medical record and confirmed with the patient during their visit on site. The following data were collected: age, sex, weight, height, type and duration of disease, current medication, previous IBD surgery, use of corticosteroids and anti TNF-α agents or other biologic drugs, smoking status, evaluation of BMD, levels of vitamin D, and presence of other relevant co-morbidities. The body mass index (BMI) was calculated as weight in kilograms divided by the square of height in meters (normal range 18–24.9 kg/m^2^).

### 2.5. Measurement of Bone Mineral Density and Vitamin D Serum Levels

BMD in the lumbar spine and femoral neck was measured by means of dual-energy X-ray absorptiometry (DXA) according to standard procedures. Values were expressed as standard deviation scores, which compare individual BMD determinations to those of young adults (T-score). Based on the World Health Organization (WHO) criteria, patients with a T-score (in the lumbar spine or femoral neck or both) between −2.5 and −1 were considered osteopenic, whereas a T-score less than −2.5 identified osteoporosis. Serum 25-hydroxyvitamin D (25(OH)D) levels <30 and <20 ng/mL were considered insufficient and deficient, respectively [25].

### 2.6. Interventions and Questionnaires

After providing their written informed consent, patients were asked to fill in a questionnaire in a waiting room and to return the form before leaving the hospital. A trained clinician was available if patients raised any questions or had any doubts, or encountered difficulties in completing it. The questionnaire did not contain identifiable data but there was a code for purposes of analysis, and patients were assured that the data would remain confidential. The questionnaire was a self-administered one, designed and constructed after a thorough literature review [21,26,27,28,29] to investigate the nutritional status in patients with IBD, and took around 30 min to be completed. Previously, the questionnaire was administered to 25 volunteers, to assess its feasibility and clarity. Then, the content validity of the survey was measured with the help of interviews and comparison between the self-administered questionnaire and clinical interviews (κ = 0.70).

The first part of the survey consisted of the visceral sensitivity index (VSI) score, used to evaluate the perception of symptoms during the day, the concern caused by the intestinal disease, and the consequence of the disease on social life [26]. It consisted of 15 questions which could be answered with a number from 1 (strongly agree) to 6 (strongly disagree). Subsequently, the numbers indicated by the patient for each item were evaluated by giving a score from 0–5, and a total score was thus reached. Since total scores ranged from 0 (no gastrointestinal (GI)-specific anxiety) to 75 (severe GI-specific anxiety), a value greater than 37.5 was chosen to indicate that the subject had an increased VSI.

The second part of the survey was represented by a food frequency questionnaire (FFQ) validated for the Italian population [27], and used to obtain information about the diet followed in the month preceding the observation and to subsequently calculate the dietary calcium intake. The FFQ was composed of 15 questions on the daily and weekly frequency of the consumption of foods and beverages with a high content of calcium most frequently used in the Italian diet: milk, milk derivatives, pasta, rice, meat, fish, eggs, legumes, fruit, vegetables, ice cream, chocolate, as well as spring water and bottled water. This section of the survey is available under Supplementary Materials.

A photographic food atlas was provided to the patient in order to evaluate the portion consumed [30]. To estimate the daily intake of calcium provided by the diet, the content of the micronutrient present in the individual foods was multiplied by the frequency and size of each food consumed [31]. Data were then compared with LARN [20], referring to the RDA, and calculated in relation to age and sex, to check whether they were adequate.

The third part of the survey was used to assess patients’ beliefs and dietary practices, through 15 questions, which investigated the potential perception that dietary habits can cause malnutrition or weakness, that a correct diet is more important than drug therapy in controlling the intestinal disease, and that nutritional behavior could represent a trigger for developing IBD. Furthermore, the questionnaire assessed whether the disease influenced the appetite and the social life of the subjects, if the patient had received nutritional advice and if they would like to receive more, if they had made changes in dietary behavior after the diagnosis of IBD, such as restriction in some foods or supplementation with nutraceutical products, and also whether they had considered alternative medicine including herbal and homeopathic products. This section of the survey is available under Supplementary Materials.

### 2.7. Statistics

The Kolmogorov–Smirnov test of normality was used to analyze the data distribution. Accordingly, continuous data were expressed as a mean plus standard deviation (SD) when normally distributed and as a median with a range if not. The socio-demographic and clinical characteristics were compared with a *t*-test (for normally distributed data) or a Mann–Whitney U test (for not normally distributed data) for continuous variables, and a chi-squared test for categorical variables. A binary logistic regression analysis was performed to identify predictors of dietary restrictions among participants. The odds ratio (OR) of having a dietary restriction, given the presence of a specific variable, was used as a measure of association and adjusted for the effect of confounding variables. The agreement between the questionnaire and reports from the medical interview, obtained during the pre-test in volunteers, was evaluated by means of the κ statistic, which measures the agreement between two observers or tests. Values range from 0 and 1, where 0 indicates an agreement expected on the basis of chance alone and 1 indicates a complete (100%) agreement. Statistical analysis was performed using the PASW statistic 18.0 software (IBM SPSS Statistics, Chicago, IL, USA). A *p*-value of less than 0.05 was considered statistically significant.

## 3. Results

### 3.1. Demographic and Clinical Characteristics of the Study Population

Ninety patients, who were consecutively admitted to the outpatient clinic with a previous diagnosis of IBD, met the eligibility criteria and accepted to fill in the questionnaire. All participants were native local residents. No patient had a history of primitive calcium metabolism imbalance, nor had undergone extensive intestinal surgery leading to a short bowel syndrome. Through the clinical interview, no special diet regimens (e.g., vegetarian or vegan, low FODMAP, or other diet with a well-defined composition) were found, which could represent potentially confounding factors in the evaluation of IBD patients’ self-prescribed dietary restrictions. Table 1 shows the demographic and clinical characteristics of the eligible IBD patients who took part in the survey. No significant difference was found between UC and CD patients in baseline characteristics.

### 3.2. Visceral Sensitivity Index and Prevalence of Dietary Restrictions

After scoring the VSI questionnaire, the mean value in the overall population was 38.8 ± 16.2, with 40.3 ± 16.5 for UC patients and 33.7 ± 14.6 for CD patients (*p* = 0.09). Considering the score of >37.5, 56 (62%) patients account for an increased visceral sensitivity, 44 of which (65%) suffered from UC and 12 (52%) from CD (*p* = 0.25).

According to the FFQ, 63 (70%) patients claimed a self-prescribed dietary restriction in at least one type of food. No difference was found between UC and CD patients (78% vs. 67%, *p* = 0.46). A clear prevalence (84%) in avoiding one or more dairy products was found. Detailed information on food exclusions is given in Table 2.

### 3.3. Risk Factors for Dietary Restrictions

At univariate analysis, dietary restrictions were significantly associated with a lower BMI (*p* = 0.009), a higher level of education (*p* = 0.002), and a higher visceral sensitivity index (*p* = 0.006). After mutual adjustment of variables with each other, a younger age (*p* = 0.02), a higher level of education (*p* = 0.007), and a higher visceral sensitivity index (*p* = 0.009) were independent risk factors for self-prescribed dietary restrictions (Table 3).

### 3.4. Calcium Intake, Vitamin D Levels, and Bone Health

According to LARN recommendations, 72 (80%) patients displayed an inadequate calcium intake, with a mean daily intake of 692.3 ± 405.8 mg. Women had a significantly lower intake compared to men (97% vs. 72%, *p* = 0.002), while no difference was found with regard to the type of disease (*p* = 0.95). The mean serum vitamin D levels in the total number of patients were 20.2 ± 7.3 ng/ml, thus falling into a condition of insufficiency according to recognized values. However, there were no statistically significant differences when comparing the mean serum vitamin D levels in patients with UC and CD (20.1 ± 7.2 vs. 18.7 ± 7.4, *p* = 0.21).

An abnormal result at DXA scan accounting for low BMD was reported in 46 (51%) patients, of which 39 (85%) suffered from osteopenia and 7 (15%) from osteoporosis. Therefore, we tested the association between the presence of low BMD and self-prescribed dietary restriction, after adjusting for the socio-demographic and disease characteristics of the patients which could affect bone health. Dietary restrictions (*p* = 0.03), and in particular avoiding dairy products (*p* = 0.001), were significant risk factors for a low BMD, along with female gender (*p* = 0.001), smoking (*p* = 0.04), and steroid abuse (*p* = 0.03), which are all acknowledged risk factors for bone derangement (Table 4).

### 3.5. Dietary Perceptions and Practices

**Figure 1:**
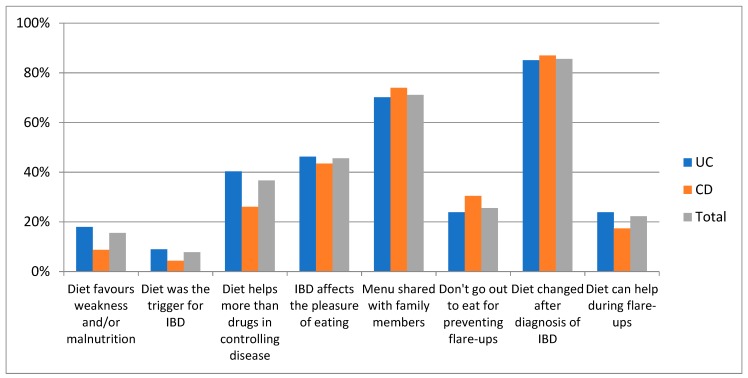
Dietary beliefs of the 90 IBD patients.

Upon analysis of the questionnaire results regarding dietary habits, almost all (86%) patients stated that their diet changed in quality and quantity of foods after IBD diagnosis. Indeed, the disease affected the appetite in 46% of patients, 8% believed that foods could have been a trigger for IBD, and 37% believed that a proper diet was more important than drugs in controlling disease behavior. Nonetheless, the majority of patients (71%) shared the same menu with their family, while only 29% followed a personalized diet. A minority (16%) of patients believed that their diet could cause weakness, micronutrient deficiency, and malnutrition and 26% of patients reported not eating out for fear of having a flare of IBD. No difference was found between UC and CD patients. These results are graphically illustrated in Figure 1.

**Figure 2:**
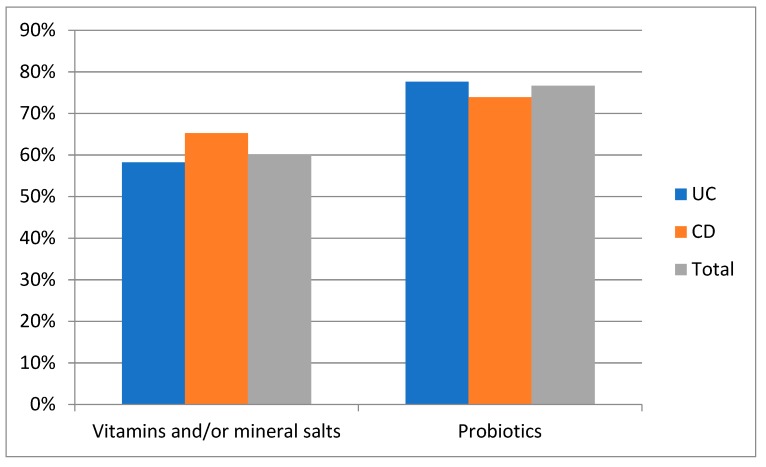
Dietary supplementations by the 90 IBD patients.

Supplementation with vitamins and/or mineral salts and probiotics was a common practice, accounting for 60% and 77%, respectively, among the patients, with no difference with respect to disease type (Figure 2). No patient declared the use of alternative therapies or herbal medicines for treating intestinal symptoms.

**Figure 3:**
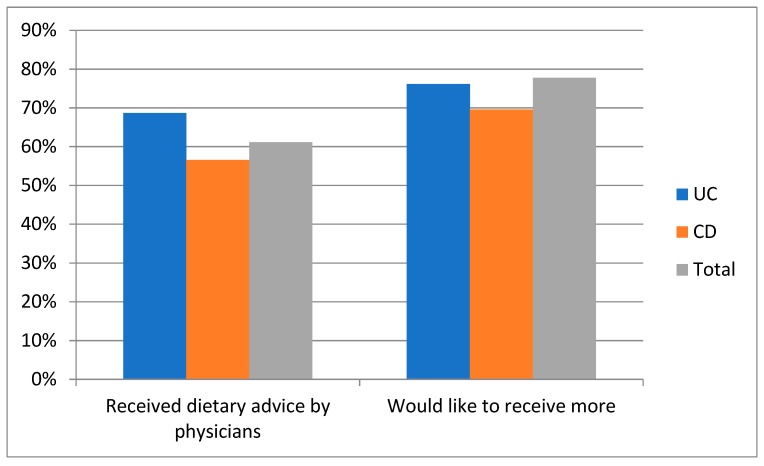
Attitude of the 90 IBD patients towards dietary advice.

More than half (61%) of patients said that they had already received information regarding diet in their disease, 69% of those with UC and 57% of those with CD. Nevertheless, 78% of the participants, of which 76% suffering from UC and 70% suffering from CD, requested more advice (Figure 3).

## 4. Discussion

Despite the serious concerns of patients about the role of food in triggering or ameliorating their intestinal disease, there are few studies dealing with patients’ beliefs and practices regarding diet in IBD [9,10]. The objective of this study was to assess how the disease affected the dietary habits of patients with IBD, and to understand if patients’ food restrictions were responsible for low bone mineralization. Therefore, we interviewed 90 patients affected by IBD regarding their dietary habits and analyzed results in relation to clinical and demographic characteristics. All patients were native local residents of Calabria, a province in southern Italy, thereby avoiding confounding factors related to dietary habits from different cultures.

The first part of the survey provided information about the VSI, with a questionnaire developed and validated to assess the gastrointestinal-specific anxiety, the behavioral response towards the fear of gastrointestinal symptoms, and the context in which these visceral sensations occur [26]. Results from the VSI questionnaire showed that the mean value was greater in UC compared with CD patients (*p* = 0.09), even if (when considering the cut-off of 37.5 chosen for increased VSI) no difference was found with regard to the disease type. Indeed, more than half (62%) of all patients showed an increased VSI, which means that they had an excessive perception of intestinal symptoms during the day, a great concern regarding their disease, and a consequent negative impact on their social life. Although the VSI questionnaire has been validated for irritable bowel syndrome (IBS), its use has been proposed as a measure of brain–gut interaction in other gastrointestinal conditions [26,32]. Indeed, a general relationship between psychological distress and gastrointestinal symptoms has been suggested and up to one-third of patients with IBD suffer from functional symptoms during remission [33,34]. Recently, a mouse model of IBD revealed that chronic colitis induced functional sequelae such as visceral hypersensitivity and increased anxiety even in the presence of a low-grade intestinal inflammation [35]. With this in mind, we decided to perform the questionnaire in our series of patients and, to the best of our knowledge, this is the first application of the VSI questionnaire to IBD patients.

With no differences between UC and CD patients, 63 (70%) participants claimed that it was their own choice to avoid at least one type of food from their diet, and among them dairy products were the most avoided category of foods. A recent online survey conducted in the Netherlands showed that a similar rate (76.5%) of IBD patients omitted foods in order to reduce disease symptoms, and 51.6% of them avoided dairy products [15]. Similarly, dairy products were found to be the most common restricted foods in several previous studies [36,37]. By analyzing the self-imposed dietary restrictions with regard to each type of food avoided, the results in our series of patients confirmed other findings in the literature [10]. This endorses the preference of IBD patients not to follow a strict diet regimen, but rather to exclude those foods considered responsible for intestinal symptoms. In support of this claim, an increased VSI was independently associated in our series with dietary restrictions. The other significant risk factors for restrictions in diet were a younger age and a higher education, but no studies on this possible relationship have been reported so far. However, having the adequate knowledge, skills, and confidence for the management of the disease has been found to strongly associate with disease outcome in a large Internet-based cohort of IBD patients [17]. An inadequate calcium intake was confirmed in 80% of the overall patients, and in 97% of women, and this is in line with previous studies showing that most IBD patients did not achieve the recommended intake of calcium with diet [36,38]. Accordingly, mean serum levels of vitamin D were found insufficient, with no difference among UC and CD patients, confirming that IBD patients display frequently low serum vitamin D concentrations [39,40]. The clinical relevance of the above-mentioned nutritional deficit involves mainly bone health, as a high rate of osteoporosis and osteopenia have been reported among IBD patients compared with the general population [41,42]. Considering that dairy products are the main source of calcium and vitamin D, we investigated the association between dietary restrictions and low bone mineralization. Results from the regression analysis identified dietary restrictions, and in particular the avoidance of dairy products, as independent risk factors for having a reduced BMD, suggesting that unnecessary food exclusion should be discouraged [23,43]. Female gender, smoking habit, and steroid abuse were also independently associated with low BMD, confirming previous findings [24,44]. On the other hand, a low BMI did not appear to be a risk factor for reduced BMD, although it has been recognized as a predictor of bone derangement [24,44].

Most of those interviewed changed their diet after diagnosis and claimed a reduction in appetite, as mentioned in other previous surveys on the dietary behavior of IBD patients [9,10]. Indeed, IBD symptoms and related medical treatments have been described as having a negative impact on dietary habits [45]. However, only 8% of our patients believed that food could be responsible for IBD development, supporting the result of 15% found by Zallot et al. [10] but different from the higher rate (57%) found elsewhere [9]. This divergence reflects the still debated role of environmental factors, including dietary habits, in the pathogenesis of IBD [46]. On the other hand, diet has been proposed as an adjunctive treatment for IBD [14,47], but only one third of our patients believed in dietary interventions for controlling disease activity. The increasing role played by food in society suggests the necessity to better profile the dietary habits in IBD patients which could impact on their quality of life. About one-fifth of our series refused to eat outdoors or to share the same menu as the family, in order to avoid a relapse. This behavior is similar in other surveys [9,10] and also in studies conducted in a pediatric IBD setting, where children rarely shared food with other family members [48]. Several reports documented that malnutrition and micronutrient deficiencies are frequently found in IBD patients, even during clinical remission, and one of the main causes is the restricted diet [49]. In our survey, only 16% of IBD patients believed that their dietary choice could promote nutritional deficiencies, and this suggests that they are not aware of such disease complications sustained by dietary restrictions. On the other hand, a large portion of those interviewed claimed the use of vitamins and/or mineral salts and probiotics, but no interest was found in herbal and homeopathic products. Indeed, the approach with complementary medicine among IBD patients is an emerging trend, while it is more consolidated in treating functional gastrointestinal symptoms [50,51].

## 5. Conclusions

Considering the growing interest of patients regarding the role of diet in IBD, and their attitude to seek information, it is important for healthcare professionals to include dietary recommendations in the routine visit, in order to avoid self-imposed restrictions with consequent adverse effects [52]. Indeed, notwithstanding the fact that more than half of the interviewees received information from their gastroenterologist, 70% stated that they would like to receive even more. Unfortunately, clinicians rarely have time to perform this kind of intervention during the routine visit; therefore, IBD patients could benefit from a separate professional figure, such as a dietician [15]. Results from our survey suggest that the management of diet in IBD represents one of the new unmet needs for these patients. Indeed, the role of diet counseling has been demonstrated to be effective against nutritional deficiencies, and further research could point out the need for an assessment of nutritional deficiencies after a dietary intervention [53,54].

More investigation is necessary to better profile the dietary behavior of IBD patients and to allow clinicians to provide adequate dietary advice.

## Figures and Tables

**Table 1:** Demographic and clinical characteristics of the inflammatory bowel disease (IBD) patients enrolled.

Variable	UC *n* = 67	CD *n* = 23	Total *n* = 90
Sex			
Male	41 (61)	13 (57)	54 (60)
Female	26 (39)	10 (43)	36 (40)
Age, years	47.5 ± 18.2	45.8 ± 15.6	47.2 ± 17.4
Disease duration, years	15.4 ± 10.5	12.4 ± 11.7	14.7 ± 10.9
BMI	24.8 ± 4.6	24.7 ± 4.2	24.8 ± 4.5
Smokers	20 (29.8)	7 (30.4)	27 (30)
Steroid-dependent	29 (43.2)	14 (60.8)	53 (58.9)
Biologic therapy	33 (49.2)	13 (56.5)	46 (51.1)
High education	35 (52)	17 (73)	52 (57)

Values are expressed as numbers (%) or means ± standard deviation. UC, ulcerative colitis; CD, Crohn’s disease; BMI, body mass index.

**Table 2:** Type of food excluded by the 63 IBD patients who claimed dietary restrictions.

	UC *n* = 45	CD *n* = 18	Total *n* = 63
Dairy Products (At least one)	38 (84)	15 (83)	53 (84)
Milk alone	12 (27)	5 (27)	17 (27)
Spicy food	28 (62)	12 (66)	40 (63)
Vegetables	29 (64)	11 (61)	40 (63)
Fibers	24 (53)	10 (55)	34 (53)
Sweet	30 (67)	14 (77)	44 (69)
Fruit	31 (68)	15 (83)	46 (73)
Legumes	25 (55)	12 (66)	37 (58)
Alcohol *	16 (35)	6 (33)	22 (35)
Coffee *	7 (15)	3 (17)	10 (16)

Values are expressed as numbers (%). * Data collected by reviewing medical records of the patients.

**Table 3:** Characteristics of the 63 patients with IBD who claimed dietary restrictions according to the food frequency questionnaire (FFQ).

Variable	Dietary Restriction Yes *n* = 63	Dietary Restriction No *n* = 27	*p*	OR (95% CI) Adjusted ^a^	*p*
Sex					
Male	34 (54)	20 (74)	0.07	1.42 (0.41–4.93)	0.57
Female	29 (46)	7 (26)			
Age, years	46.8 ± 17.7	48.0 ± 16.9	0.76	1.05 (1.08–1.11)	**0.02**
BMI	24.2 ± 4.5	26.8 ± 3.4	**0.009**	0.88 (0.75–1.03)	0.13
UC CD	45 (71.4) 18 (28.6)	22 (81.4) 5 (18.5)	0.38	1.93 (0.49–7.57)	0.34
Disease duration, years	13.6 ± 10	17.1 ± 12	0.16	0.96 (0.90–1.03)	0.32
Steroid-dependent	27 (42.9)	16 (52.3)	0.15	0.37 (0.08–1.54)	0.17
High education	43 (68.3)	9 (33.3)	**0.002**	7.07 (1.72–18.83)	**0.007**
Smokers	16 (25)	10 (37)	0.26	3.44 (0.93–12.07)	0.06
High VSI	45 (71.4)	11 (40.7)	**0.006**	5.22 (1.51–8.01)	**0.009**
Biologic therapy	29 (46)	17 (63)	0.14	0.55 (0.13–2.20)	0.40

BMI, body mass index; CI, confidence interval; MVA, multivariate analysis; OR, odds ratio; SD, standard deviation; UC, ulcerative colitis; CD, Crohn’s disease; VSI, visceral sensitivity index. Values are numbers (%), mean ± SD or median with range as indicated; means were compared using a Student’s *t*-test when data were normally distributed and a Mann–Whitney U test when data were not normally distributed, and proportions were determined using a chi-squared test. ORs with 95% CI in brackets are given. Bold text indicates a statistically significant difference with a *p*-value less than 0.05. ^a^ All variables except age, disease duration, and BMI entered MVA analysis as categorical variables.

**Table 4:** Characteristics of the patients according to low bone mineral density (BMD).

Variable	Low BMD *n* = 46	Normal BMD *n* = 44	*p*	OR (95% CI) Adjusted ^a^	*p*
Sex					
Male	18 (39)	36 (81)	**0.000**	12.81 (4.78–25.43)	**0.001**
Female	28 (61)	8 (19)			
Age, years	46.6 ± 16.5	47.6 ± 18.4	0.78	0.98 (0.87–1.15)	0.75
BMI	24.2 ± 4.6	25.7 ± 3.9	0.08	0.91 (0.76–1.17)	0.25
Type of disease					
UC	35 (52)	32 (72)	0.90	1.45 (0.98–5.36)	0.62
CD	11 (48)	12 (27)			
Disease duration, years	15.6 ± 10.8	16.3 ± 10.9	0.40	1.05 (0.91–4.23)	0.11
Dietary restriction	35 (76)	28 (63)	0.19	1.16 (1.03–1.85)	**0.03**
Avoiding dairy products	29 (63)	13 (30)	**0.001**	11.6 (4.33–28.71)	**0.001**
Vitamin D levels	20.2 ± 7.4	19.2 ± 7.1	0.50	0.91 (0.87–1.16)	0.78
Inadequate calcium intake	38 (83)	34 (77)	0.24	2.26 (0.45–5.26)	0.31
Smokers	15 (32)	11 (25)	0.28	1.67 (1.32–4.23)	**0.04**
Steroid-dependent	25 (54)	18 (40)	0.20	4.08 (1.14–7.56)	**0.03**

BMI, body mass index; CI, confidence interval; MVA, multivariate analysis; OR, odds ratio; SD, standard deviation; UC, ulcerative colitis; CD, Crohn’s disease; VSI, visceral sensitivity index. Values are numbers (%), mean ± SD or median with range as indicated; means were compared using a Student’s *t*-test when data were normally distributed and a Mann–Whitney U test when data were not normally distributed, and proportions were determined using a chi-squared test. ORs with 95% CI in brackets are given. Bold text indicates a statistically significant difference with a *p*-value less than 0.05. ^a^ All variables except age, disease duration, and BMI entered MVA analysis as categorical variables.

## Supplementary Materials

The following are available online at https://www.mdpi.com/1010-660X/55/8/507/s1, Table S1: Questionnaire on dietary habits (Part three of the survey), Table S2: Food Frequency Questionnaire (part two of the survey).
