# Supplementary material for: Self-Prescribed Dietary Restrictions are Common in Inflammatory Bowel Disease Patients and Are Associated with Low Bone Mineralization
Source: Medicina (Kaunas). 2019 Aug 20;55(8):507. doi: 10.3390/medicina55080507 (PMC6722983; doi:10.3390/medicina55080507)
Supplement: Supplementary file 1 [file medicina-55-00507-s001.pdf]

Table S1. Questionnaire on dietary habits (Part three of the survey).

**Through administration of this questionnaire, we may evaluate beliefs and dietary practices among IBD patients. Please, mark the appropriate box with an X.**
1. Do you believe that your dietary habits can cause weakness, malnutrition or nutrient deficiencies? Yes ☐ No ☐
2. Do you take vitamin supplements and / or mineral salts? Yes ☐ No ☐
3. Do you take alternative therapies or herbal medicines for your intestinal disease? Yes ☐ No ☐
4. Do you take food supplements? Yes ☐ No ☐
5. Do you take probiotic supplements? Yes ☐ No ☐
6. Do you believe that food was the trigger for your intestinal disease? Yes ☐ No ☐
7. Do you think that eating habits have a more important role than drugs in controlling your intestinal disease? Yes ☐ No ☐
8. Do you think your intestinal disease affects appetite and eating pleasure? Yes ☐ No ☐
9. Do you share the same menu as other family members? Yes ☐ No ☐
10. Do you refuse to eat out for fear of having a relapse? Yes ☐ No ☐
11. Did you change your diet after diagnosing your IBD? Yes ☐ No ☐
12. Do you tend to avoid certain foods? Yes ☐ No ☐
13. Do some foods improve symptoms during a recurrence of your IBD? Yes ☐ No ☐
14. Have you received advice on your diet from medical personnel? Yes ☐ No ☐
15. Would you like to receive more nutritional advice? Yes ☐ No ☐

Table S2. Food Frequency Questionnaire (part two of the survey) [Montomoli M. Gonnelli S. Giacchi M.et al. Validation of a food frequency questionnaire for nutritional calcium intake assessment in Italian women. Eur J Clin Nutr. 2002;56(1);21–30].

1 – DO YOU DRINK **MILK** (except SOIA milk)? Yes ☐ No ☐
how much? **A**-one glass (100ml) ☐ **B**-one mug (250 ml) ☐ **C**-two mugs (500 ml) ☐
how many times per week? ....

2 – DO YOU EAT **YOGURT**? Yes ☐ No ☐
how much? **A**-one pottle (125g) ☐ **B**-two pottles (250 g) ☐ **C**-one wrapping of 500g ☐
how many times per week? ....

3 – DO YOU EAT **CHEESE**? Yes ☐ No ☐
how many times per week? ....

<u>which kind?:</u>

3.1 HARD CHEESE (Parmigiano, Grana, …)? Yes ☐ No ☐
how much? **A**-small serving (40g) ☐ **B**-medium serving (80g) ☐ **C**-large serving (120g) ☐
how many times per week? ....

3.2 SEMI-HARD CHEESE (Emmenthal, Provolone, ...)?Yes ☐ No ☐
how much? **A**-small serving (40g) ☐ **B**-medium serving (80g) ☐ **C**-large serving (120g) ☐
how many times per week? ....

3.3 SOFT CHEESE (Mozzarellla, Stracchino, …)? Yes ☐ No ☐
how much? **A**-small serving (40g) ☐ **B**-medium serving (80g) ☐ **C**-large serving (120g) ☐
how many times per week? ....

3.4 BUTTER MILK CURD? Yes ☐ No ☐
FROM SHEEP? ☐ FROM COW? ☐
how much? **A**-small serving (50g) ☐ **B**-medium serving (100g) ☐ **C**-large serving (200g) ☐
how many times per week? ....

4 – DO YOU EAT **PASTA** or **RICE**? Yes ☐ No ☐
how much? **A**-small serving (50g) ☐ **B**-medium serving (100g) ☐ **C**-large serving (150g) ☐
how many times per week? ....

- AND WITH HOW MANY TEASPOONS OF **PARMIGIANO/GRANA**?
A-none ☐ **B**-1 teaspoon (5g) ☐ **C**-2 teaspoons (10g) ☐ **D**-3 teaspoons (15g) ☐

5 – DO YOU EAT **BREAD** or similar (crackers, bread-stick, …)? Yes ☐ No ☐
how much per day? **A**-100 g (=2 rosette) ☐ **B**-200 g ☐ **C**-300 g ☐
how many times per week?..........

6 – DO YOU EAT **POTATOES**? Yes □ No □
how many? **A**- small serving (200g=2 medium potatoes) □
B-medium serving (400g) □
C-large serving (600g) □
How many times per week?..........

7 – DO YOU EAT **MEAT** OR **FISH**? Yes □ No □
how much? **A**-small serving (100g) □ **B**-medium serving (150g) □ **C**-large serving (200g) □
how many times per week? ....

8 – DO YOU EAT **EGGS**? Yes □ No □
how many eggs per week? ....

9 – DO YOU EAT **LEGUMES** (beans, peas,…)? Yes □ No □
how many? **A**-small serving (80 g cooked – 1/3 of a tin) □
B-medium serving (150 g cooked –half tin) □
C-large serving (250 g a cooked– a tin) □
How many times per week? ....

10 – DO YOU EAT **VEGETABLES**? Yes □ No □
how many? **A**-small serving (100 g) □ **B**-medium serving (200 g) □ **C**-large serving (300 g) □
how many times per week? ....

11 – DO YOU EAT **FRESH FRUITS**? Yes □ No □
how many per week? ....

12 – DO YOU EAT **ICE CREAM with milk** (except sorbet)? Yes □ No □
how much? **A**-small serving (50 g) □ **B**-medium serving (100 g) □ **C**-large serving (150 g) □
how many per week? ....

13 – DO YOU EAT **milk or white CHOCOLATE**? Yes □ No □
how much? **A**-small serving (25 g) □ **B**-medium serving (50 g) □ **C**-large serving (100 g) □
how many times per week? ....

14 – DO YOU DRINK **TAP'S WATER**? Yes □ No □
how much per day? 0,500 l □ 0,750 l □ 1l □ 1,5 l □ 2 l □ 2,5l □
how many times per week?………….

15 – DO YOU DRINK **CALCIUM RICH MINERAL WATER** Yes □ No □
how much per day? 0,500 l □ 0,750 l □ 1l □ 1,5 l □ 2 l □ 2,5l □
how many times per week?………
